# Understanding Animal Group-Size Distributions

**DOI:** 10.1371/journal.pone.0023438

**Published:** 2011-08-30

**Authors:** Michael Griesser, Qi Ma, Simone Webber, Katharine Bowgen, David J. T. Sumpter

**Affiliations:** 1 Population Biology and Conservation Biology, Department of Ecology and Evolution, Uppsala University, Uppsala, Sweden; 2 Department of Ecology, Swedish University of Agricultural Sciences, Uppsala, Sweden; 3 Institute for Evolution and Ecology, University of Bern, Bern, Switzerland; 4 Department of Mathematics, Uppsala University, Uppsala, Sweden; 5 Centre for Ornithology, University of Birmingham, Edgbaston, Birmingham, United Kingdom; Hungarian Academy of Sciences, Hungary

## Abstract

One of the most striking aspects of animal groups is their remarkable variation in size, both within and between species. While a number of mechanistic models have been proposed to explain this variation, there are few comprehensive datasets against which these models have been tested. In particular, we only vaguely understand how environmental factors and behavioral activities affect group-size distributions. Here we use observations of House sparrows (*Passer domesticus*) to investigate the factors determining group-size distribution. Over a wide range of conditions, we observed that animal group sizes followed a single parameter distribution known as the logarithmic distribution. This single parameter is the mean group size experienced by a randomly chosen individual (including the individual itself). For sparrows, the experienced mean group size, and hence the distribution, was affected by four factors: morning temperature, place, behavior and the degree of food spillage. Our results further indicate that the sparrows regulate the mean group size they experience, either by groups splitting more or merging less when local densities are high. We suggest that the mean experienced group size provides a simple but general tool for assessing the ecology and evolution of grouping.

## Introduction

Groups of animals are seen engaged in behaviors as diverse as social foraging [1], [2], predator detection [3], [4], and navigation [5], [6]. There are a whole range of costs and benefits to individuals in groups and understanding why and how groups form is fundamental to understanding social behaviors [7]. One of the most basic questions about these groups concerns their size distribution. Group sizes of animals often range over several orders of magnitude, even when these different sized groups contain members of the same species living in similar environments [8]. What determines these group sizes and why there is such a variation in their size?

The theoretical study of animal group sizes can be approached both in terms of function and mechanism [9], [10]. The first mechanistic models emphasized the use of the negative binomial distribution for animal group-size distributions [11], [12], [13]. Under the negative binomial distribution, the probability of observing a group of size *N* is given by
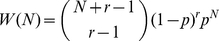
(equation1)Okubo predicted that group sizes should follow a geometric distribution, which is a specific case of the negative binomial with *r* = 1, and he presented a number of empirical cases where this relationship held [14]. The Poisson distribution is also a single parameter special case of the negative binomial obtained by letting *r* go to infinity while holding the distribution mean constant.

While the negative binomial distribution does fit some datasets, the most striking aspect of many empirical observations is the large variance and long tail of group-size distributions (i.e. the occurrence of very large groups) [15]. Even the geometric distribution, which maximizes the variance of the negative binomial distribution does not capture the extent of this variation, with group sizes often ranging over several orders of magnitude. A number of alternative mathematical models have tried to explain the mechanisms through which group-size variation arises [14], [16], [17], [18], [19], [20], [21]. For example, Bonabeau and Dagorn proposed a model for animal grouping based on a single assumption: if groups meet they always merge to form a larger group [17], [18]. Their model predicts power law distributions of group sizes, which appeared consistent with some observational data of fish and mammals. In particular, they proposed that truncated power laws such that the probability of finding a group of size *N* is
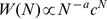
(equation2)where *a*>0 and 0*<c*<1 are constants, should be wide spread in nature. The parameter *a* determines the slope of the power law and *c* determines the point at which the power law is truncated. Similar results were found by Sjöberg [8], although they used a slightly different truncation scheme.

Recently, Niwa proposed a simple distribution of animal grouping and tested it against fish schooling data [20], [21]. He predicted that the probability *W*(*N*) of observing a group of size *N* is proportional to
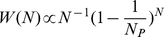
(equation3)where 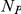 is the expected group size experienced by a randomly chosen individual including the individual itself (see also [22]). The key model parameter 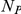 can be estimated directly from observations, i.e. 
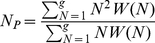
(equation4)where *g* is the maximum observed group size, and W(N) is the observed fraction of groups of size *N*. 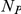
is generally larger than the observed mean group size, 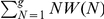, since randomly chosen individuals are more likely to be in larger groups. A detailed derivation and discussion of equation 3, which is known as the **logarithmic distribution**, can be found in [23].

The logarithmic distribution provides a simple, single parameter model of group-size distribution. There are several reasons to expect it to be of practical use [23]. Firstly, both Niwa [20], [21] as well as Gueron and Levin [16] give first principles derivations of this model based on simple rules for how animals leave and join groups. Secondly, there is a very natural relationship between the model's parameter and a naturally observable feature, i.e. the average group size experienced by an individual. Finally, and most importantly, Niwa showed that group-size distributions for six different fish species were all accurately fitted by equation (3) [20]. This was a remarkable observation, simply by determining 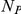 for a particular species, Niwa was able to predict the entire distribution of group sizes. Finally, the logarithmic distribution is a special case of both the truncated power law in equation 2, with *a = *1 and *c* = (1-1/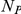), and the negative binomial distribution as *r* goes to zero [23].

While Niwa's and other truncated power law models provide elegant descriptions of group-size distributions, they do not address the functional or ultimate questions about why groups form. Niwa's derivation of the logarithmic distribution was purely mechanistic. It postulated that if groups merge and split in a certain way we expect a particular relationship between the expected group size experienced by an individual and the overall group-size distribution. This mechanistic approach can be contrasted with a functional approach that calculates the costs and benefits of group membership to find an optimal group size. Living in groups provides benefits in terms of increased safety from predators, information transfer and energy conservation, but costs in terms of increased rate of disease transmission or competition over limited resources [7], [24]. Sibly further argued that, even when we know the benefits and costs of grouping, isolated individuals can gain by joining a group even when that group is larger than optimal [25]. Few empirical studies have established a clear relationship between the mean group size and costs and benefits to an individual as a result of group membership, although see [24], [26] for notable exceptions.

Understanding why groups have certain typical sizes and distributions and how these change with external factors is central to understanding the social dynamics of groups. Jovani et al. [27] have recently looked at how group-size distribution is affected by population density, transitioning from a power law to a truncated power law when the population increases. Here, we provide a comprehensive investigation into the role of environmental factors and behaviors on group-size distributions of House sparrows (*Passer domesticus*). House sparrows in a rural valley in southern France were chosen as a study system due to their tendency to form non-familial groups outside of the breeding season. This small-sized passerine generally lives in close proximity with humans and benefits from feeding on food spills that result from agricultural practices [28]. During the breeding season sparrows breed in pairs and defend the area surrounding their nesting site against conspecifics. Outside the breeding season sparrows form groups that roost and forage together. While most pairs split after the breeding season and re-mate during winter with a different individual, some pairs remain together between breeding seasons [29]. The natural variation in the environment at our study site was used to identify the factors which determine not only average group size, but also the distribution of group sizes. In doing so, we aimed at linking the mechanistic explanations of group-size distribution in sparrows to the ultimate reasons why animals form groups.

## Results

**Figure 1:**
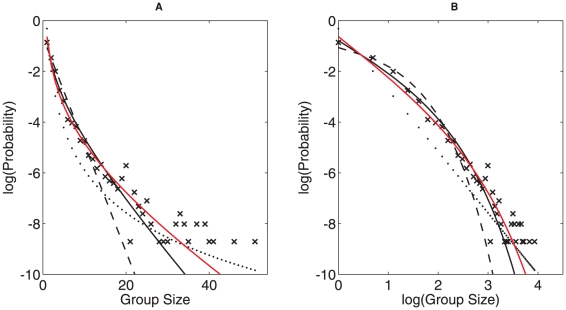
Distribution of group sizes for all observations. Comparison of the empirical data (x) with N_mean_ = 2.9 and 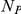 = 7.3, a power law (dotted line) with α = 2.42, geometric distribution (dashed line) with p = 0.35, logarithmic distribution (solid line) with 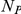 = 6.4, truncated power law by MLE (almost congruent with the line for logarithmic distribution and thus not displayed) with a = 0.99, c = 0.84 and truncated power law by minimizing χ^2^ value (red line) with a = 1.45, c = 0.91 on a semi-log (A) and a log-log plot (B). Number of observations: *n* = 6070.

**Figure 2:**
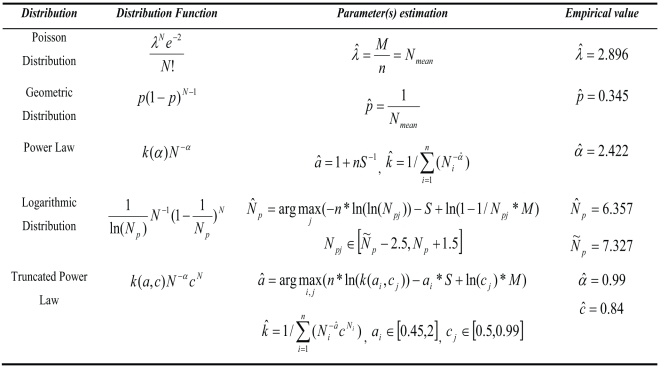
Maximum likelihood estimation for optional models. Where *n* = 6070 is the sample size, i.e. total number of groups observed; N_i_ (i = 1,…,n) are all the observations, i.e. number of individuals in group i; and for convenience, we denote 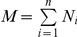 and 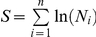. For the logarithmic distribution, the normalization factor is 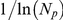 (detailed derivation can be found in 23]). 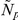 is the expected group size experienced by a randomly chosen individual, calculated directly from the data according to equation 4. We first calculated 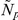 and then searched the neighborhood of 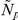 to get the 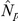 which maximizes the likelihood function.

Sparrow group-size distribution over all distributions varied between 1 and 46 (fig. 1) while the average group size experienced by an individual was 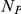 = 7.33. We fitted four alternative single parameter models to the data: a Poisson distribution (conditioned on group sizes being greater than or equal to one), a geometric distribution, a power law and the logarithmic distribution (equation 3). Figure 1 shows the best fit of each model, while table 1 gives fitting statistics and the estimated parameter values are given in figure 2. The best fit of all the models was provided by the logarithmic distribution (with 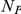 = 6.36). The Poisson distribution provided a very poor fit to the data (AICδ>5000) and is not shown in figure 1. The geometric distribution fit well in the middle of the distribution but not in the tail (AICδ = 637). The empirical distribution was not a straight line in a log-log plot and as such was poorly fit by a pure power law (AICδ = 2489). Although a χ^2^ test would lead us to reject all these theoretical distributions as perfectly describing the data, the logarithmic distribution provides the best single parameter description of the data.

In terms of AIC, the logarithmic distribution also outperformed both of the alternative two parameter models: the negative binomial distribution (equation 1) and a truncated power law (equation 2). The best fit for the negative binomial distribution was consistent with that predicted by geometric distribution (i.e.
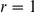
_)_. The maximum likelihood estimated parameters of the truncated power law were *a* = 0.99 and *c = *0.84. These values are almost identical to those given by the logarithmic distribution (i.e. *a* = 1 and *c* = 1-1/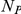 = 1-1/6.36  = 0.84). It is thus unsurprising that the AICδ = 2 and the logarithmic distribution has a better fit when the number of parameters are accounted for. Since all the alternative models were rejected by χ^2^ test, and the truncated power law was the second best model after the logarithmic distribution by AICδ, we tried to fit the data with truncated power law which minimizes χ^2^ value using the same class division as stated above. The best χ^2^ value we got was χ^2^  = 14 when a = 1.45 and c = 0.91, this result passes χ^2^ test, but meanwhile it has a higher AICδ = 191 compared to the truncated power law fit by MLE (AICδ = 2) and a lower 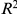 = 0.93.

An alternative approach is to check the multiplicative binned data in log-log plot. A previous study showed that multiplicative binned log-log plot was better for empirical fat-tailed group size data [27]. We used this technique to compare different alternative models. Table 1 summarizes the five different criteria we used for model fit. Overall different fitting methods show little qualitative difference in terms of their predictions about which model fits the data best. The logarithmic is the best of all single parameter models and better or only marginally worse than the truncated power law.

**Table 1:** Comparison of five statistics for proposed models.

Distribution	AIC Rank (δ AIC)	R^2^ Rank	χ^2^_ Rank_	R^2^ (log-log) Rank	χ^2^ (log-log) Rank	Number of parameters
Logarithmic (N_p_ = 6.36 estimated by MLE)	1 (0)	2 (0.985)	2 (89)	2 (0.848)	1 (22)	1
Truncated Power Law (MLE)	2 (2)	2 (0.985)	3 (93)	1 (0.849)	2 (27)	2
Logarithmic (N_p_ = 7.33 calculated from data)	3 (39)	1 (0.986)	4 (134)	3 (0.818)	6 (104)	1
Truncated Power Law (minχ^2^)	4 (191)	5 (0.927)	1 (14)	4 (0.789)	4 (40)	2
Geometric (Negative Binomial)	5 (637)	4 (0.965)	6 (>5000)	6 (0.442)	3 (37)	1
Power Law	6 (2489)	7 (0.525)	5 (943)	5 (0.751)	5 (96)	1
Poisson	7 (>5000)	6 (0.619)	7(>10000)	–	–	1

Models are ranked in order of their AIC (Akaike Information Criterion) scores, and other ranking are given along with values for corresponding statistics.

**Figure 3:**
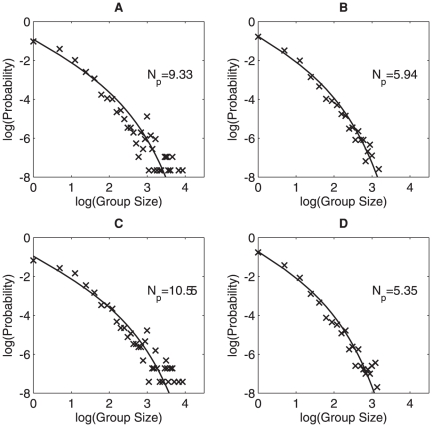
Effect of temperature and place on group-size distribution. Group-size distribution for initial morning temperatures below 6°C (number of observations, *n* = 2113) (A), initial morning temperatures above 6°C (*n* = 3957) (B), for groups located in hedges or on food (*n* = 1668) (C) and for groups located elsewhere (*n* = 4402) (D).

**Figure 4:**
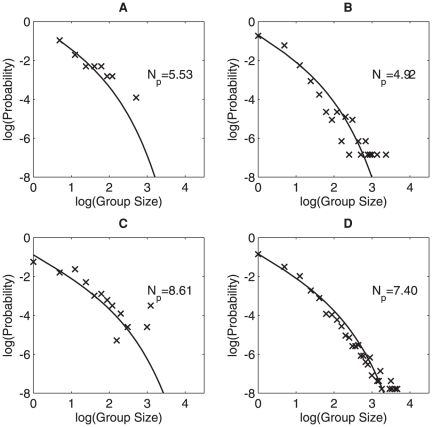
Effect of behavior on group–size distribution. Group-size distribution for individuals who are fighting (number of observations, *n* = 50) (A), flying (*n* = 942) (B), foraging (*n* = 199) (C) and perching (*n* = 4797) (D). Fighting distributions are adjusted to account for the fact that group sizes must be equal to or greater than 2.

Although the logarithmic distribution with 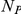
estimated by MLE was the best fit for our data, the same distribution with N_P_ estimated directly from data (i.e. using equation 4) also resulted in a good fit. It even has a higher 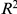 value than the fit with MLE. It is thus rather straightforward and convenient to use 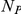
from the data without losing much goodness of fit. We therefore used 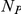
directly from the data in the logarithmic distribution to assess the influence of environmental factors (fig. 3 and 4).

To assess further which environmental factors affected the group-size distribution we first inverse transformed the data and used a generalized linear mixed model (table 2). The inverse transform reflected the exponential tail of the distribution of group sizes. Three factors were found to affect group size: morning temperature on the day of the observation, place and behavior while the degree of food spillage had a marginal influence on group size. Factors that might reflect predation risk (e.g. the number of cats) or disturbances (e.g. presence of humans) had no significant effect on group sizes.

**Table 2:** Generalized linear mixed model (GLIMMIX module in SAS 9.1; exponential error function; Type III Tests of Fixed Effects) showing the effect of independent model terms on House sparrow group sizes (n = 6067 groups) in 36 locations.

Effect	Num DF	Den DF	F Value	P Value
Place ^a^	5	5479	15.93	<.0001
Activity ^b^	4	5479	10.82	<.0001
Morning temperature ^c^	1	5479	30.48	<.0001
Degree of food spillage ^d^	3	5479	2.35	0.07
Number of cats	1	5479	1.03	0.31
Disturbance ^e^	3	5476	1.76	0.15
Food sources ^f^	1	5479	0.96	0.33
Livestock diversity ^g^	1	5478	0.65	0.42
Distance nearest location ^h^	1	5479	1.79	0.18
Weather ^i^	2	5477	0.20	0.82

The effect of non-significant terms was estimated by adding them individually in to the final model. Minute of scan was nested within site and date and added as random factor into the model to control for the effect of repeated observations within a given site.

a =  Place: air, ground, hedge, tree, house and wires, food

b =  Activity: fight, fly, forage, perch

c =  Temperature in degree C

d =  Food spillage: locations without food spillage (i.e. maize, chicken food, grains, manure, hay), minor food spillage, medium degree of food spillage in several places, large degree of food spillage in the whole location

e =  Disturbance occurred during sampling (i.e. passing by car, human)

f =  Number of different crops, animal foods stored at the site

g = Number of different stock in each site (i.e. horses, cows, sheep, pigs, chicken)

h =  Distance to next location in m

I =  Weather during the observation: foggy, strong wind or rain, normal weather (i.e. no fog, strong wind or rain).

Sparrows aggregated in larger groups on cold days than on warmer days. To illustrate this effect we split the data set into two halves in respect to morning temperatures. On cold days with low morning temperature, the average group size experienced by an individual was more than 50% larger (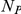 = 9.33) than on days with warm morning temperatures (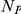 = 5.94). Niwa predicted that a change in 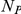 will result in a shift in the point at which group-size distribution changes from a power law to exponential.

Such a shift is seen in the data when we plot group size distributions below and above 6°C separately (fig. 3a,b). A similar change of 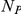
also occurred when we use place and behavior (see table 2 for the divisions) as criteria to divide the data into different subgroups, the comparison of group size distribution for groups in different places is shown in figure 3c,d and figure 4 compares group size distributions for groups engaged in different behaviors. Sparrow groups that were located on houses or in the air were significantly smaller than groups that were sitting on hedges or on a food source. Accordingly, flying sparrows were in smaller groups than sparrows that were foraging. When perching, the main activity of sparrows outside the breeding season, the mean experienced group size was between those seen when flying and foraging.

**Figure 5:**
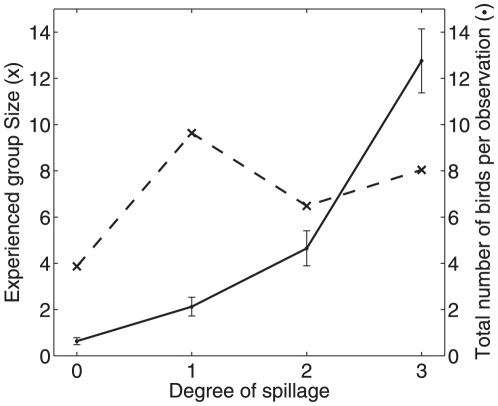
Effect of food spillage. The average group size experienced by an individual (x) and the average total number of birds per observation (•) for different food spillage levels. For the average total number of birds per observation, we took the mean of each 15-minute observation interval and averaged all the means in the same food spillage level. The error bar shows the standard error of the means.

Do the sparrows actively regulate their group sizes or is it simply determined by the density of the birds in a particular area? This question goes to the heart of stable group size theory. If group size is simply proportional to the number of birds available to form a group then this would suggest that the birds' aggregations result only from a common attraction to particular features in the environment, rather than an active regulation in response to other individuals. In particular, Niwa [21] predicts that if there is active aggregation then
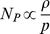
(equation6)where *ρ* is the population density and *p* is the probability per time step that a group splits apart. We can investigate this question by looking at the effect of food spillage on group-size distributions. Figure 5 reveals that the mean total number of birds per observation increases with degree of food spillage (Wilcoxon rank-sum test for different mean number of birds, no spillage vs. spillage level 1 has *z* = 5.81 , *P*<0.001 , similar tests show statistical difference between all spillage levels). However figure 5 also shows that while sparrows aggregated in smaller groups in locations with no food spillage, average group size experienced by the individual did not increase with larger amounts of food spillage. Assuming equation 6 holds, we thus predict that splitting rate increases with group size to counterbalance the increase in local population density.

## Discussion

Our results support the robustness of the logarithmic distribution for describing animal group-sizes [20], [21]. Unlike the fish catch data used by Niwa, we used data sampled from wild birds in a non-intrusive form of observation. While the match between data and the logarithmic distribution is not perfect, it has a large explanatory power. The differences between the model and data are seen for groups of 2 to 4 birds which might be explained by sparrows leaving and joining groups in established pairs [29]. The addition of an extra parameter in the truncated power law did not significantly improve the fit. The logarithmic distribution (equation 3) with either 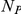 = 6.36 given by MLE or 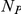 = 7.33 directly from the data (equation 4) is a very good fit by the 5 statistics we calculated. Given the single parameter 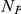
has a natural biological interpretation, and is readily estimated from data, we would propose the logarithmic distribution as a simple but general law for animal grouping.

The relationship established by Niwa between mean group size experienced by an individual and the distribution of group sizes observed holds for groups of sparrows. The underlying biology of a species determines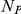, but once we have estimated 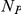 we can then determine the group-size distribution of this species in full. This observation could apply over a wide range of species, and prove a useful tool in characterizing interspecies differences and differences between environments for a single species. We have thus shown how Niwa's model can be applied to study functional aspects of group-size distribution.


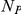
 allows us to assess how animals change their rate of leaving and joining groups in response to environmental differences. Individuals were more likely to form groups when foraging, which might reflect the use of social information when looking for food [7], [24], or safer foraging conditions in a larger group [9]. As food spillage increased and food became easier to find, the sparrows regulated the mean group size they experienced by splitting more often when local densities were higher.

The environmental and social factors do not affect the shape of the distribution of group sizes, but instead the parameter 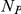 varies with different factors. Indeed, when we aggregate all of the data in figure 1 we get similar distributions as in figures 3 and 4 albeit with different 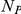. The mathematical reason for this scaling is that equation 3 predicts an identical slope of 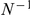 for small and medium sized *N*, independent of the truncation in the distribution determined by 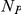. Furthermore, the probability of observing a group of size above that of the truncation at 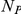 decreases exponentially fast, so that if we aggregate two such distributions the rate of decrease lies somewhere in between that of the two aggregated distributions. As a result, we see for example that in figure 3a, 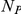 = 9.3 for low temperatures and 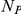 = 5.9 for higher temperatures, but in the amalgamated data (fig. 1) 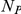 = 7.3 lies roughly half way between these two values.

Our study suggests that animals combine the group size they experience with environmental factors to make grouping decisions. Earlier studies of animal grouping have emphasized the use of optimal and stable group sizes in the functional interpretation of data. Optimal and stable points of a distribution are obtained by finding the maximum or a particular extreme of group-size distributions. Niwa's model and our data show that even if individuals change their rate of leaving and joining groups as a function of environmental and social cues, we still expect to observe a wide distribution of different group sizes. As a result 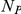 is a far simpler and more informative tool for assessing the behavioral ecology of grouping than optimal or stable points on a group-size distribution. By better understanding the mechanisms that produce group-size distribution we are better able to assess the functional aspects of grouping.

## Materials and Methods

### Study Site

We collected data for this study in a population of House Sparrows between November 2007 and March 2008 in Lantabat, about 40 km to the east of Biarritz, Southern France. The community of Lantabat is located in a well confined valley that is surrounded by a mountain ridge on three sides. The landscape structure is characterized by small scale agriculture, in particular by traditional sheep herding on small meadows as well as cattle production. Maize is the only cereal crop cultivated in the valley and is done so on a small scale for livestock use. The majority of farm houses have traditional open maize storages where whole maize cobs are stored in an outdoor frame, that the birds take advantage of for foraging.

The settlements in the valley range from single houses (≈ 50) to three larger hamlets with up to 30 houses. For our sparrow surveys, we selected 36 settlements that were at least 100 m apart from each other (mean distance between settlements  = 252 m, min  = 110 m, max  = 850 m). The size of the surveyed settlements varied between one and 30 buildings (mean  = 4.6).

### Data Collection

Preliminary surveys showed that sparrows were not active on days with high wind or rain levels and thus data were not collected under these conditions. Each of the three observers surveyed the same settlements and used always the same observation location within the settlements. We choose different routes through the study site to sample data in the same location at different times during the day. To assess group sizes in the different locations, we counted the number of groups in each location 10 times and recorded the group size, place and activity (see below for detailed definitions).

We used extensive observations before the onset of data collection to come up with a meaningful definition of a group [9]. An individual belonged to a group if it was at a maximum of 4 m away from the nearest sparrow. Upon arrival birds either (i) joined an already present group (close contact, individuals intermingle), (ii) actively avoided an already present group (and landed further than 4 m away), or (iii) did not join any other individuals independent of the context (i.e. foraging, perching). This suggests that 4 m seems to be a biologically meaningful distance to separate groups, although it is not possible to exclude that this distance varies between contexts or individuals.

For the places we used the following categories:

air  =  sparrow flying

ground  =  sparrow located on ground, in a field or a meadow

hedge  =  sparrow located in a hedge (branches provide cover down to the ground)

tree  = sparrow located in a tree (lowest part of tree without cover)

house  =  sparrow located on a building (house, barn, church, derelict building)

wire  =  sparrow located on a wire, power line or phone line

For each group, we assessed the main activity of the group members. In cases where sparrows in a group were displaying more than one activity, we chose the activity in which most members were engaged. For the assessment of the activities we used the following categories:

fight  =  sparrows interacting aggressively either on the ground or in the air, see [30] for definition of aggression.

fly  =  sparrow flying

forage  =  sparrow foraging or handling food

perch  =  sparrow perched.

In cases where the sparrows were hiding in dense vegetation making it difficult to assess their behavior, we classified their activity as out of sight.

We used a scan-sampling protocol [31] where we instantaneously scanned the location for sparrow groups once per minute during a 15 min period with the help of binoculars. Upon arrival to a location we used the first 2–5 min to locate sparrows and count group sizes before starting data collection. We used the time between scans to monitor changes in group sizes and locations to be able to scan accurately again at the onset of the next minute. If the sparrows were located in gutters, under the roof or in dense hedges, group sizes might have been underestimated. In the three large settlements with more than five buildings, all three observers counted the sparrows simultaneously from three different locations with a non-overlapping observation range. While this sampling protocol did not allow counting the maximum number of individuals present in a location, it gave a rough proxy for the maximum number of sparrows in a location. Moreover, it allowed for sampling of group sizes and group-size distributions in a comparable manner in all locations.

To assess the effect of environmental variation between the locations on group sizes, we surveyed the whole study site and assessed if the settlement contained an active farm, a partially active farm (farmers that were not actively farming large numbers of livestock but still had a few chicken and/or ducks on their farm), or if there was no active farm present. We also assessed the number of livestock, the degree of animal food spillage categorized as locations without food spillage (i.e. locations without farms and thus no spillage of maize, chicken food, grains, manure, hay on the ground), locations with minor food spillage (locations with few animals which are fed (chickens, ducks) but no livestock), locations with intermediate food spillage (farms with livestock some food spillage in a few places), and farms with livestock with a large degree of food spillage in the whole location. In addition we also counted the number of cats present in each location as they can prey upon sparrows.

During the observations, we noted all disturbances (presence of a predator, human passing through the surveyed perimeter, vehicles (car, tractor, HGV)) and the weather conditions. On each observation day we recorded the morning temperature before starting the surveys.

### Fitting of Distributions

We used maximum likelihood estimation (MLE) to obtain the parameters for all the proposed models. In fitting the distributions we used all available data, i.e. all of the one minute observations within each 15 minute period. The decision to use all the data is based on the assumption that the group size distribution is in equilibrium, whereby each leaving or joining event takes the group from one point in the distribution to another. In any case, group composition changed rapidly, so there was seldom replication of group sizes from one minute to the next and large groups rapidly split in to smaller groups. Although (as we state above) sampling biases are likely to be small, we note that any potential bias would occur for larger group sizes, thus weakening the fit of logarithimic or power law distributions and strengthening the fit of the negative binomial distribution.

Estimation results and details are shown in table 1. We also estimated *r* and *p* for the negative binomial distribution NB(r,p) (i.e. equation 1). However, MLE gave 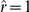, which is identical with the geometric distribution and the result is therefore omitted from figure 2. For each of the proposed distributions - Poisson, geometric, power law, logarithmic (equation 3) and the truncated power law (equation 2) - we calculated five statistics to quantify the difference between the observed 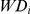 fraction of groups in size class *i* and the theoretical probability density 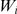. The first three statistics are
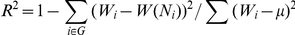


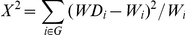
and
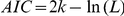
In calculating 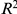 the set of size classes consisted of all group sizes observed in the empirical data. For χ^2^, we set 10 size classes, the first class consisted of groups with size from 1 to 4, the second was from 5 to 8, the third was from 9 to 12, …, and so on for the first nine classes. The last class contained all groups whose size is no smaller than 37. The third statistic, AIC, is the Akaike information criteria [32], [33], which takes both the fit and number of parameters estimated into account. It is a test between models, an important criterion for model selection. The AIC is based on the likelihood function L, which is defined as 
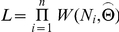
where 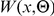 is the proposed probability density function of group size 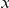 under parameter 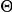. Since AIC is calculated to compare the goodness of fit of all the proposed models, it is sufficient to use
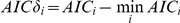
as the index of goodness of fit. Here 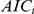 denotes the AIC value for model i.

For high skewed distribution like exponential and power law distribution, usually the error for data fitting is not normally distributed, errors in the tail are underestimated by normal scale, therefore we also calculated a further two 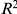 and χ2 values for data plotted on a log-log scale. The formula for these is given by
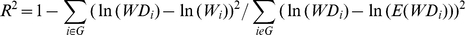
and
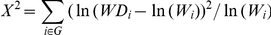



### Effect of environmental variables on group sizes

Given that the individual group sizes followed a negative exponential curve, we used the reciprocal transformation. This transformation resulted in group sizes that followed an exponential function. We used the GLIMMIX module in SAS 9.1 (SAS institute, Cary, North Carolina) to analyze the data. We tested for the effect of the environmental variables, temperature, cluster size against an exponential error distribution. We included minute of the sampling event nested within location identity and date as random effects into the model. This allowed us to control for the repeated sampling on each observation and the nested data structure. We added in all models all explanatory terms of interest and possible interactions, and subsequently dropped all terms that did not influence the explanatory power of the model (a priori α = 0.05).

## References

[1] Barnard CJ, Sibly RM (1981). Producers And Scroungers - A General-Model And Its Application To Captive Flocks Of House Sparrows.. Animal Behaviour.

[2] Giraldeau LA, Beauchamp G (1999). Food exploitation: searching for the optimal joining policy.. Trends in Ecology & Evolution.

[3] Treherne JE, Foster WA (1981). Group transmissin of predator avoidance behaviour ina marine insect: the Trafalgar effect.. Animal Behaviour.

[4] Hamilton WD (1971). Geometry for Selfish Herd.. Journal of Theoretical Biology.

[5] Biro D, Sumpter DJT, Meade J, Guilford T (2006). From compromise to leadership in pigeon homing.. Current Biology.

[6] Weimerskirch H, Martin J, Clerquin Y, Alexandre P, Jiraskova S (2001). Energy saving in flight formation - Pelicans flying in a ‘V’ can glide for extended periods using the other birds' air streams.. Nature.

[7] Krause J, Ruxton GD (2002). Living in groups.

[8] Sjöberg M, Albrectsen B, Hjalten J (2000). Truncated power laws: a tool for understanding aggregation patterns in animals?. Ecology Letters.

[9] Krause J, Ruxton GD (2002). Living in groups..

[10] Sumpter DJT (2010). Collective Animal Behavior..

[11] Caraco T (1980). Stochastic dynamics of avian foraging flocks.. American Naturalist.

[12] Cohen JE (1972). Markov Population Processes as Models of Primate Social and Population Dynamics.. Theoretical Population Biology.

[13] Morgan BJT (1976). Stochastic-Models of Grouping Changes.. Advances in Applied Probability.

[14] Okubo A (1986). Dynamical aspects of animal grouping.. Advances in Biophysics.

[15] Gerard JF, Bideau E, Maublanc ML, Loisel P, Marchal C (2002). Herd size in large herbivores: Encoded in the individual or emergent?. Biological Bulletin.

[16] Gueron S, Levin SA (1995). The Dynamics Of Group Formation.. Mathematical Biosciences.

[17] Bonabeau E, Dagorn L (1995). Possible Universality In The Size Distribution Of Fish Schools.. Physical Review E.

[18] Bonabeau E, Dagorn L, Freon P (1999). Scaling in animal group-size distributions.. Proceedings of the National Academy of Sciences of the United States of America.

[19] Niwa HS (1998). School size statistics of fish.. Journal of Theoretical Biology.

[20] Niwa HS (2003). Power-law versus exponential distributions of animal group sizes.. Journal of Theoretical Biology.

[21] Niwa HS (2004). Space-irrelevant scaling law for fish school sizes.. Journal of Theoretical Biology.

[22] Jarman P (1982). Prospects for interspecific comparison in sociobiology. Current Problems in Sociobiology..

[23] Ma Q, Johansson A, Sumpter DJT (2011). A first principles derivation of animal group size distributions.. Journal of Theoretical Biology.

[24] Brown CR, Brown MB (1996). Coloniality in the Cliff Swallow..

[25] Sibly RM (1983). Optimal group-size is unstable.. Animal Behaviour.

[26] Aviles L, Tufino P (1998). Colony size and individual fitness in the social spider Anelosimus eximius.. American Naturalist.

[27] Jovani R, Serrano D, Ursua E, Tella JL (2008). Truncated Power Laws Reveal a Link between Low-Level Behavioral Processes and Grouping Patterns in a Colonial Bird.. PLoS One.

[28] Barnard CJ (1980). Factors affecting flock size mean and variance in a winter population of house sparrows (Passer domesticus L).. Behaviour.

[29] Anderson TR (2006). Biology of the ubiquitous house sparrow : from genes to populations..

[30] Johnson CA, Grant JWA, Giraldeau LA (2004). The effect of patch size and competitor number on aggression among foraging house sparrows.. Behavioral Ecology.

[31] Altmann S (1974). Observational study of behaviour: sampling methods.. Behaviour.

[32] Akaike H (1974). New Look at Statistical-Model Identification.. Ieee Transactions on Automatic Control.

[33] Edwards AM (2008). Using likelihood to test for Levy flight search patterns and for general power-law distributions in nature.. Journal of Animal Ecology.

